# Psychometric validation of the Chinese-adapted eczema behavior checklist extent scale in pediatric atopic dermatitis patients

**DOI:** 10.3389/falgy.2025.1667971

**Published:** 2025-11-13

**Authors:** Jian Sun, Linna Li, Chang Qi, Xue Tian

**Affiliations:** Department of Dermatology and Venereology, the First Affiliated Hospital of Jinzhou Medical University, Jinzhou, Liaoning, China

**Keywords:** eczema behavior checklist, behavior problems, atopic dermatitis, psychometric properties, pediatric patients

## Abstract

**Background:**

Pediatric atopic dermatitis (AD) demonstrate significantly higher rates of both general behavioral problems and condition-specific maladaptive behaviors. These behavioral challenges often interfere with parental treatment adherence and compromise disease management effectiveness. The Eczema Behavior Checklist Extent Scale (EBC-ES), initially developed and validated in Australia, represents the first psychometric tool specifically designed to evaluate AD-specific behavioral problems in children. However, its cross-cultural applicability and validation in Chinese populations remain unexplored.

**Objective:**

This study aimed to culturally adapt and validate the Chinese version of the EBC-ES for assessing AD-specific behavioral problems in pediatric patients across China.

**Methods:**

The Chinese EBC-ES utilized Brislin's validated back-translation protocol for cultural adaptation. This cross-sectional study recruited 674 parents (mean age 35.5 ± 4.7 years, range 24–49) of 3–10-year-old children (mean 5.9 ± 1.8) with physician-diagnosed AD. The sample comprised 369 boys (54.7%) and 305 girls (45.3%). Participants completed the Chinese EBC-ES and the Eyberg Child Behavior Inventory-Intensity scale (ECBI-IS). Psychometric evaluation included exploratory and confirmatory factor analyses (EFA, CFA) to assess construct validity, content validity indices (CVI), internal consistency (Cronbach's *α*, McDonald's *ω*), split-half reliability, and test-retest reliability.

**Results:**

The final 24-item Chinese EBC-ES demonstrated a stable three-factor structure (eigenvalues >1), accounting for 80.44% of the total variance. The Kaiser-Meyer-Olkin measure confirmed sampling adequacy (KMO = 0.942), and Bartlett's test supported factorability (*χ*² = 14,091.013; *p* < 0.001). CFA indicated excellent model fit: chi-square degree of freedom (*χ*²/df) = 2.855, root mean square error of approximation (RMSEA) = 0.075, standardized root mean square residual (SRMR) = 0.041. Comparative Fit Index (CFI) = 0.948, Tucker Lewis Index (TLI) = 0.942, Normed Fit Index (NFI) = 0.923, and Incremental Fit Index (IFI) = 0.948. The scale showed strong content validity (CVI = 0.96), high internal consistency (*α*=0.968, *ω* = 0.987), excellent test-retest reliability (*r* = 0.969), and satisfactory split-half reliability (*r* = 0.895).

**Conclusion:**

The Chinese version of the EBC-ES demonstrates robust psychometric properties, confirming its reliability and validity for AD-specific child behavioral problems in both clinical practice and research settings within Chinese populations.

## Introduction

Atopic dermatitis (AD), a chronic relapsing inflammatory dermatosis characterized by recurrent eczematous plaques and severe pruritus, imposes substantial biopsychosocial burdens on the global pediatric population (1). With a worldwide prevalence of 15%–20% in pediatric populations (2), China reports 12.94% prevalence among children aged 1–7 years (3). The disease typically emerges in infancy, with 60%–70% of cases developing symptoms before age 1 (4, 5), and 70%–95% exhibiting clinical manifestations by 5 years (6). AD management requires multimodal strategies addressing skin barrier dysfunction, immune dysregulation, and environmental triggers, reflecting its complex pathophysiology. However, high treatment burden frequently leads to poor adherence, exacerbating disease control challenges.

### Challenges in managing pediatric atopic dermatitis: impact of treatment complexity and child behavior on adherence and outcomes

The management of pediatric AD necessitates multimodal interventions comprising daily emollients, medicated baths, topical anti-inflammatory agents, systemic therapies, and allergen avoidance (7–9). However, treatment complexity often leads to poor adherence, particularly in moderate-to-severe cases (10, 11), requiring collaboration among caregivers, healthcare providers, and children to manage behavioral challenges.

This complexity directly contributes to suboptimal adherence, which stems from multiple factors: complex treatment regimens requiring precise execution, time-intensive medication routines, financial constraints, unfavorable sensory characteristics of topical therapies (e.g., stickiness, odor), long-term safety concerns, and limited parental understanding of disease mechanisms and treatment principles (8). Clinical evidence indicates adherence declines from 40% at week 1%–32% by week 8 even with cost-free treatments (12).

Critically, child behavioral resistance poses a significant barrier to adherence through disrupted treatment execution and caregiver frustration. This contributes to poorer clinical outcomes, including increased disease severity, sleep disturbances, and reduced quality of life (13). Evidence indicates that 66% of caregivers encounter treatment noncooperation in children with AD, largely driven by medication avoidance behaviors (e.g., refusal of topical therapies or aversion to corticosteroid-induced sensory effects). These behaviors critically undermine adherence, necessitating targeted family behavioral interventions to enhance public health outcomes (14).

Suboptimal adherence to pediatric AD treatment regimens-including bathing protocols, topical applications, and systemic medications-substantially further elevates caregiver burden, particularly in terms of psychological distress and physical strain (15). Population-based studies reveal significant variation in treatment acceptance rates, ranging from 80.3% for basic emollients (16) to 26.7% for targeted therapies like crisaborole 2% ointment (17). Most critically, treatment resistance in children (evidenced by refusal behaviors and physical avoidance) predicts poorer clinical outcomes, with resistant cases showing less than 50% symptom improvement compared to adherent counterparts (18).

Children with AD demonstrate dual behavioral burdens: AD-specific challenges and elevated rates of generalized behavioral problems compared to healthy peers, both correlating with disease severity (19, 20–22). Consequently, caregivers report heightened distress, perceived incompetence in behavioral management, and increased utilization of conflict-avoidant disciplinary strategies (23, 24). Evidence-based parenting interventions are therefore critical, yet rigorous studies on pediatric AD-specific behaviors remain limited. Although general scales [e.g., Eyberg Child Behavior Inventory (ECBI)] are commonly used to assess behavioral issues in AD, standardized instruments designed for AD-specific behaviors in children are lacking.

### Eczema behavior checklist extent scale

The Eczema Behavior Checklist Extent scale (EBC-ES), initially developed and validated by Mitchell et al. (15) in Australia, is the first psychometrically robust instrument specifically designed to assess AD-specific behavioral problems in pediatric populations. This 25-item scale consists of three domains: treatment-related behaviors, impact-related behaviors, and symptom-related behaviors. Initial validation (15) demonstrated adequate psychometric properties (including reliability and validity), supporting its use in evaluating AD-specific behaviors in clinical and research settings.

To date, however, the EBC-ES lacks cross-cultural validation outside Australia. Future research should validate the scale's psychometric characteristics and applicability for assessing AD-specific behavioral problems in Chinese children.

### Purpose of the present study

The EBC-ES currently lacks validation in Chinese pediatric populations with atopic dermatitis (AD), limiting its clinical applicability for evaluating AD-specific behavioral problems in children. The introduction of a validated Chinese version of this instrument would provide a standardized and reliable measure for evaluating AD-specific behavioral problems in children, particularly within China's unique sociocultural context. This culturally adapted tool instrument interventions for children with AD.

This study establishes three core objectives: (1) determining the psychometric validity and reliability of the cross-culturally adapted Chinese EBC-ES in pediatric AD patients through combined Exploratory and Confirmatory Factor Analyses; (2) quantifying its convergent validity with the Eyberg Child Behavior Inventory-Intensity Scale (ECBI-IS); and (3) identifying sociodemographic disparities in scale performance across diverse population subgroups.

## Methods

### Study design and participants

This cross-sectional investigation was conducted at the First Affiliated Hospital of Jinzhou Medical University, China, between October 2023 and October 2024. The sample size was determined using a standard of 5–10 participants per questionnaire item, as used in previous validation studies at our institution (25), to ensure adequate statistical power and model stability. A total of 674 eligible parents were recruited and completed anonymous questionnaires. The study received ethical approval from the Institutional Review Board of Jinzhou Medical University (Approval ID: JZMULL2023105), with written informed consent obtained from all participants' legal guardians. Inclusion criteria comprised parents of children aged 3–10 years with a physician-diagnosed AD based on the U.K. Working Party's Diagnostic Criteria (Williams criteria). All participants completed the Chinese versions of the EBC-ES and the ECBI-IS (26). Sociodemographic data were collected, including child/parental sex, child comorbidities, residence, parental role, parental education level, and household income. Test-retest reliability was evaluated in a randomly selected subsample (*n* = 50), who recompleted the scales after a standardized 2-week interval.

### Instruments

#### Questionnaire on general demographic characteristics

A demographic questionnaire was developed through the study objectives and literature review, collecting data on child/parental age and sex, child comorbidities, place of residence, parental role, parental education level, household income, and clinical characteristics.

#### Eczema behaviour checklist extent scale (EBC-ES)

The EBC-ES is a validated parent-report tool specifically designed to measure AD-specific behavioral problems in children and adolescents. This instrument was originally developed by Mitchell et al. (15) for pediatric behavioral assessment in AD populations. Psychometric validation of the original 25-item scale defined three clinically relevant domains through factor analysis: treatment-related behaviors (10 items), impact-related behaviors (7 items), and symptom-related behaviors (7 items). Participants assessed behavior severity during the preceding 28-day period using a 7-point Likert scale with defined behavioral descriptors: 1 (not at all) to 7 (very much). The AD-Specific Behavioral Problem Score (range: 25–175) was derived through summation of all items, with elevated scores indicating heightened behavioral impairment severity. The scale exhibited high internal consistency in its original validation study (Cronbach's *α* = 0.96). Previous studies establish its reliability, validity, and sensitivity for assessing AD-specific behavioral problems in pediatric populations (15).

#### Eyberg child behavior inventory intensity scale (ECBI-IS)

The ECBI-IS is a 36-item parent-reported measure originally developed by Eyberg et al. (26). It assesses disruptive behaviors in children aged 2–16 years on a 7-point Likert scale (1 = never to 7 = always), with the total score indicating severity of behavioral problems. Validated as both a screening and outcome tool in clinical and research settings, its psychometric properties demonstrate strong reliability and brevity (26). To establish test-retest reliability, participants completed the scale twice at a 2-week interval. Test-retest reliability was assessed by readministering the scale to participants after a two-week interval.

### Procedures

#### Translation and culture adaptation of the scale

The original instrument was cross-culturally adapted in accordance with Brislin's translation framework (27), consistent with the standardized methodology used in previous validation studies conducted at our institution (28, 29). Formal copyright permission was obtained from Professor A. E. Mitchell. First, the EBC-ES was independently translated from English into Chinese by two qualified professionals: a medical expert and a psychologist. This process minimized linguistic and cultural biases and prioritized conceptual equivalence. Second, translators and researchers collaboratively reviewed the initial Chinese translation, resolving linguistic and conceptual discrepancies through iterative discussions to reach a consensus version. Subsequently, to assess cultural and conceptual equivalence, the Chinese draft was independently back-translated into English by two bilingual experts who were blinded to the original scale. Finally, the research team and original translators systematically compared the original scale, Chinese draft, and back-translated versions. Items with inconsistencies underwent linguistic validation and cultural adaptation to ensure cultural relevance and conceptual accuracy for the Chinese context. Pre-implementation cognitive interviews (*n* = 20 parents) assessed the translated scale's readability and interpretability, confirming high comprehensibility and feasible completion duration (mean = 4 min). The final Chinese adaptation of EBC-ES was produced after rigorous translation procedures.

#### Statistical analysis

All quantitative analyses were performed using IBM SPSS Statistics (version 27.0; IBM Corp., Armonk, NY, USA) for descriptive and comparative assessments, while confirmatory factor analysis and path modeling were implemented through AMOS 24.0. Normally distributed continuous variables, confirmed by skewness (absolute values ≤1.0) and kurtosis (absolute values ≤2.0) metrics (30), were expressed as mean ± standard deviation. Between-group comparisons of EBC-ES Chinese version scores across demographic and clinical characteristics employed parametric tests (independent samples t-test for binary variables; one-way ANOVA for multi-category variables), with a predefined significance threshold of *α* = 0.05 (two-tailed) (31, 32).

CFA was employed to validate the measurement model of the Chinese version EBC-ES. Model fit evaluation employed multiple criteria: (1) Absolute fit indices, specifically the ratio of chi-square to degrees of freedom (*χ*²/df), the root mean square error of approximation (RMSEA), and the standardized root mean square residual (SRMR); (2) Incremental fit indices, encompassing the comparative fit index (CFI), Tucker–Lewis index (TLI), normed fit index (NFI), and incremental fit index (IFI) (33–36). Convergent validity was evaluated through AVE values, and composite reliability was determined using CR values. Discriminant validity was examined using the heterotrait—monotrait ratio (HTMT). Maximum reliability (Max R) was computed to account for potential cross-loadings (32, 37). Reliability assessment incorporated measures of internal consistency, including Cronbach's alpha coefficient, McDonald's omega coefficient, and split-half reliability coefficients, as well as temporal stability, determined through test-retest reliability coefficients.

## Validity analysis

### Construct validity

#### Exploratory factor analysis (EFA) and confirmatory factor analysis (CFA)

Structural validity, defined as the congruence between an instrument's dimensional structure and its theoretical foundations (36), was examined via sequential psychometric validation phases: initial exploratory factor analysis (EFA) succeeded by confirmatory factor analysis (CFA). Using computer-generated randomization, 674 participants were allocated into two independent cohorts: EFA validation cohort (n_1_ = 337) and CFA confirmation cohort (*n*_2_ = 337). For Sample 1 (*n*_1_ = 337), principal component analysis (PCA) employing Varimax rotation was conducted on the Chinese EBC-ES to assess its factor structure. Construct validity verification encompassed three key metrics: Component loadings, Eigenvalues (quantifying variance per factor), and Cumulative variance. Data factorability was validated through psychometric criteria: Kaiser-Meyer-Olkin (KMO) measure >0.60 and significant Bartlett's sphericity test (*p* < 0.001), meeting established standards (38, 39). PCA utilizing varimax rotation was conducted for factor extraction. The analysis retained components demonstrating eigenvalues exceeding 1.0, with variables maintaining factor loadings of ≥0.40 incorporated into the final factor structure (40). Factor retention was determined based on parallel analysis, a minimum cumulative variance of 60%, and scree plot examination. CFA was subsequently conducted on Sample 2 (*n*_2_ = 337) to psychometrically validate the EFA results and assess the proposed structural model.Model fit evaluation employed established indices: (1) *χ*²/df; (2) RMSEA; (3) SRMR; (4) CFI; (5) TLI; (6) NFI; (7) IFI. Consistent with established criteria (41–43), model fit was deemed acceptable when: *χ*²/df < 3, RMSEA < 0.08, SRMR < 0.08, and CFI/TLI/NFI/IFI > 0.90. To psychometrically validate the CFA-derived model, five key coefficients were quantified: (1) CR; (2) AVE; (3) MSV; (4) Max H; (5) (HTMT) (32, 37). Convergent validity was psychometrically confirmed with the following dual criteria: AVE > 0.50 and CR > 0.70 (44, 45). Discriminant validity was confirmed, as all AVE values surpassed their respective MSV measures (46). Construct reliability was established, with both CR and Max R (H) values exceeding 0.70 (47, 48). Discriminant validity was established, as all HTMT estimates remained under the recommended 0.85 threshold (49).

#### Content validity

Content validity of the Chinese EBC-ES was evaluated through two established metrics: the Item-Content Validity Index (I-CVI) for individual item analysis and the Scale-Content Validity Index (S-CVI/Ave) for overall scale assessment (50). Six independent medical experts evaluated the content relevance of individual items to their corresponding theoretical dimensions using a 4-point Likert scale (1 = no correlation; 4 = very strong correlation). Based on conventional psychometric standards (51, 52), validity thresholds were set at I-CVI > 0.78 per item and S-CVI/Ave > 0.90 for the full instrument.

#### Criterion validity

Criterion validity was examined via Pearson correlation analysis between Chinese EBC-ES and ECBI-IS total scores. Following established psychometric standards (41), evidence thresholds were: strong correlation (*r* ≥ 0.70), moderate correlation (0.30 ≤ *r* < 0.70).

### Reliability analysis

#### Internal consistency reliability

Internal consistency reliability was assessed using three indices: Cronbach's alpha coefficient, McDonald's omega coefficient, and split-half reliability coefficients. Following established psychometric benchmarks (53, 54): *α* ≥ 0.70 (acceptable), *ω* ≥ 0.80 (excellent).

#### Test-retest reliability

Test-retest reliability was evaluated in a randomly selected subsample (*n* = 50) after a 2-week interval. Pearson's correlation coefficient was computed to measure consistency between assessments, with a correlation coefficient ≥0.70 indicating adequate reliability based on established psychometric standards (52).

## Results

### Sociodemographic characteristics and differential analysis

The final analytic sample included 674 of the initially recruited 697 individuals (96.7%). As detailed in Table 1, the cohort comprised children with atopic dermatitis and their parents, encompassing a spectrum of socioeconomic and clinical characteristics. Differential analysis identified several sociodemographic factors significantly associated with EBC-ES scores.

**Table 1:** Socio-demographic characteristics of participants (***N*** = 674).

Characteristics	Groups	Participants [*n* (%)]	Mean (SD)	*P*-value
Child's age (years)			5.94 ± 1.83	
Parent's age (years)			35.54 ± 4.75	
Child sex				0.021
	Male	369 (54.7%)	77.66 ± 24.01	
	Female	305 (45.3%)	73.20 ± 25.90	
Parental role				0.030
	Mother	549 (81.5%)	76.64 ± 25.84	
	Father	125 (18.5%)	71.28 ± 20.20	
Parental education level				0.467
	Primary or less	93 (13.8%)	77.36 ± 24.95	
	Secondary or higher	581 (86.2%)	75.20 ± 25.54	
Comorbidities				0.877
	Asthma	237 (35.2%)	75.08 ± 21.61	
	Allergic rhinitis	344 (51.0%)	73.09 ± 28.27	
	Allergy/anaphylaxis	205 (30.4%)	77.41 ± 22.15	
Place of residence				0.225
	Urban areas	551 (81.8%)	74.85 ± 25.30	
	Rural areas	123 (18.2%)	77.93 ± 24.76	
Household income				0.042
	<$20,000	387 (57.4%)	77.42 ± 24.36	
	$20,000–$40,000	183 (27.2%)	74.58 ± 23.52	
	>$40,000	104 (15.4%)	70.32 ± 27.54	

Caregivers of male children reported significantly higher burden scores than those of female children (*p* = 0.021). Furthermore, maternal-reported scores were significantly higher than paternal-reported scores (*p* = 0.030). Additionally, household income demonstrated a clear inverse relationship with burden scores (*p* = 0.042). In contrast, parental education level, comorbidities, and place of residence were not significantly associated with scores in this sample (all *p* > 0.05).

### Descriptive statistics of scale scores

Table 2 presents the psychometric properties of the Chinese EBC-ES, with items ranked by severity (mean ± SD). Normality was confirmed, with all skewness (range: −1 to 1) and kurtosis (range: −2 to 2) values within acceptable thresholds for normality. The total scale score (73.06 ± 24.95) revealed that 17/25 items (68%) exceeded the clinical threshold (mean scores >3), indicating moderate-to-severe behavioral disturbances in pediatric AD. Symptom-related behaviors showed the greatest clinical severity (mean scores >4), particularly pruritus-associated scratching, skin barrier dysfunction, and sleep disruption. Treatment-related behaviors demonstrated moderate severity (mean scores: 3–4), comprising both therapeutic non-adherence (e.g., resistance to bathing or topical therapy due to discomfort) and treatment-associated emotional dysregulation (e.g., tantrums during procedures). Impact-related behaviors were the least clinically severe domain (mean scores <3).

**Table 2:** EBC-ES items ranked by mean score with skewness and kurtosis values (*N* = 674).

Items	Mean (SD)	Skewness	Kurtosis
19 Complains about eczema	4.38 (1.63)	0.318	−0.945
20 Complains about eczema symptoms	4.36 (1.78)	0.342	−1.007
23 Whinges or whines about eczema	4.34 (1.50)	0.284	−0.989
22 Complains about wanting to scratch	4.31 (1.67)	0.336	−1.004
25 Damages their skin further by scratching	4.29 (1.69)	0.332	−0.977
24 Has trouble settling at bedtime or overnight	4.25 (1.56)	0.404	−0.918
21 Complains about being itchy	4.05 (1.60)	0.367	−0.840
4 Complains that baths or creams “sting”	3.95 (1.71)	0.522	−0.534
2 Complains about having creams applied	3.84 (1.55)	0.557	−0.411
3 Complains about the “feel” of baths or creams	3.76 (1.63)	0.555	−0.506
5 Argues about having a bath or applying creams	3.41 (1.40)	0.428	−0.629
12 Refuses to apply creams when eczema flares up	3.32 (1.24)	0.746	−0.343
7 Refuses or resists having a bath or applying creams	3.31 (1.47)	0.478	−0.337
1 Complains about having a bath	3.27 (1.39)	0.496	−0.354
6 Behaves disruptively when bathing or applying creams	3.22 (1.37)	0.616	−0.165
8 Throws a tantrum about having a bath or applying creams	3.03 (1.31)	0.664	−0.235
9 Tries to remove creams after application	3.01 (1.42)	0.680	−0.404
10 Forgets to apply their creams	2.81 (0.59)	0.470	1.427
17 Becomes concerned or anxious when eczema flares up	1.81 (0.53)	0.489	0.531
15 Frequently visits the nurse at school	1.71 (0.46)	−0.906	−1.187
11 Applies creams incorrectly	1.68 (0.56)	0.440	1.437
18 Refuses to participate in activities	1.65 (0.42)	0.360	1.259
13 Complains about going to the doctor	1.61 (0.50)	0.639	0.910
14 Refuses to go to school	1.55 (0.48)	0.966	0.557
16 Frequently comes home from school	1.48(0.64)	0.105	0.152

### Item analyze

The initial 25-item EBC-ES demonstrated exceptional internal consistency (Cronbach's *α* = 0.971). However, item-level analysis revealed that Item 15 had suboptimal psychometric properties, with a low corrected item-total correlation [*r* = 0.19, below the recommended threshold of >0.30 (55)] and inadequate content validity (I-CVI = 0.407, below the acceptable cutoff of ≥0.78) (19) as rated by six independent experts. After removing Item 15, the final 24-item scale achieved higher internal consistency (Cronbach's *α* = 0.975), confirming the enhanced psychometric properties of the revised version (Table 3).

**Table 3:** Cronbach alpha if the item is deleted (*N* = 674).

Item	Cronbach alpha if the item was deleted	Corrected Item-Total Correlation
1	0.749	0.899
2	0.748	0.888
3	0.747	0.877
4	0.747	0.830
5	0.748	0.824
6	0.749	0.883
7	0.749	0.897
8	0.749	0.864
9	0.748	0.885
10	0.763	0.589
11	0.762	0.586
12	0.749	0.834
13	0.763	0.581
14	0.764	0.553
15	0.975	0.190
16	0.764	0.566
17	0.762	0.527
18	0.748	0.621
19	0.748	0.849
20	0.749	0.842
21	0.748	0.797
22	0.748	0.821
23	0.748	0.829
24	0.748	0.832
25	0.749	0.845

## Validity analysis

### Construct validity

#### Exploratory factor analysis (EFA)

Data suitability for factor analysis was confirmed on Sample 1 (*n*_1_ = 337), with Bartlett's test of sphericity showing significant correlations (*χ*² = 14,091.013, *p* < 0.001) and KMO measure indicating excellent sampling adequacy (KMO = 0.942, >0.6 threshold). Principal component analysis (PCA) with varimax rotation extracted three factors (eigenvalues >1) explaining 80.44% total variance (37.489%, 27.600%, and 15.354%, respectively). All 24 items demonstrated strong factor loadings (range: 0.616–0.871). Detailed results are shown in Table 4.

**Table 4:** Factor loadings of the exploratory factor analysis with 24 items (*n*_1_* =* 337).

EBC Item number	Component		
	Eczema treatment behaviors	Eczema impact behaviors	Eczema symptoms behaviors
1	0.829		
2	0.839		
3	0.832		
4	0.871		
5	0.830		
6	0.807		
7	0.836		
8	0.788		
9	0.833		
10		0.696	
11		0.836	
12	0.869		
13		0.647	
14		0.616	
15		0.718	
16		0.619	
17		0.815	
18			0.855
19			0.843
20			0.827
21			0.851
22			0.847
23			0.796
24			0.830

**Figure 1:**
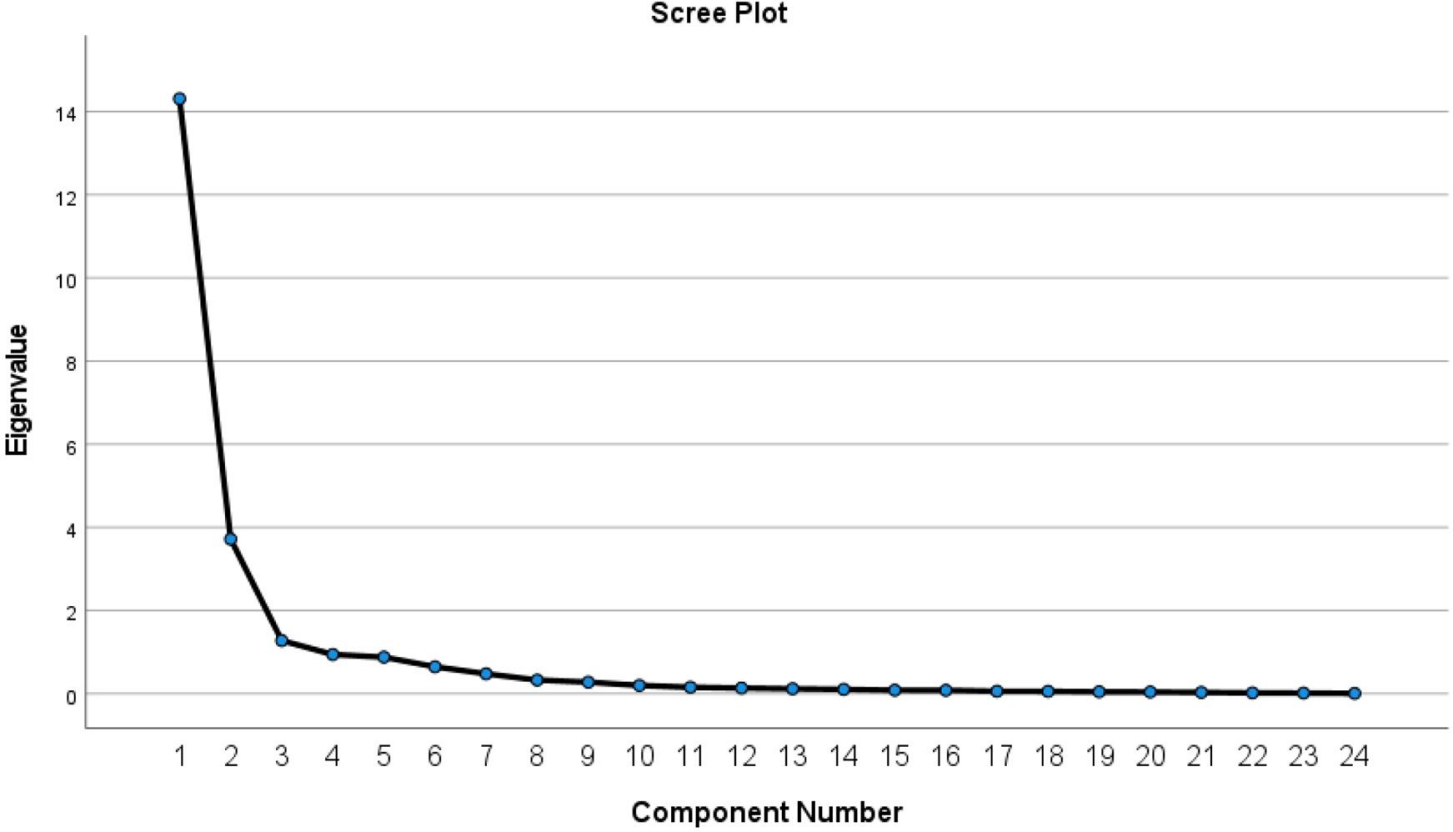
Scree plot from exploratory factor analysis of the Chinese EBC-ES (*n*_1_ = 337).

The scree plot further validated the three-factor structure, with a discernible leveling off observed after the third eigenvalue component. The scree plot is presented in Figure 1.

#### Confirmatory factor analysis (CFA)

CFA was conducted on Sample 2 (*n*_2_ = 337) to validate the three-factor structure identified through EFA. The model demonstrated good fit: *χ*²/df = 2.855, RMSEA = 0.075, SRMR = 0.041, CFI = 0.948, TLI = 0.942, NFI = 0.923, and IFI = 0.948. All indices met prespecified benchmarks, with full results detailed in Table 5.

**Table 5:** Goodness-of-fit indexes of the three-factor structural model for the Chinese revision of EBC-ES (*n*_2_ = 337).

Model	*χ*^2^/df	RMSEA	SRMR	CFI	TLI	NFI	IFI
Factor model	2.855	0.075	0.041	0.948	0.942	0.923	0.948

χ^2^, chi-square; df, degrees of freedom; RMSEA, root mean square error of approximation; SRMR, standardized root mean residual; CFI, comparative fit index; TLI, Tucker–Lewis index; NFI, normed fit index; IFI, incremental fit index.

**Figure 2:**
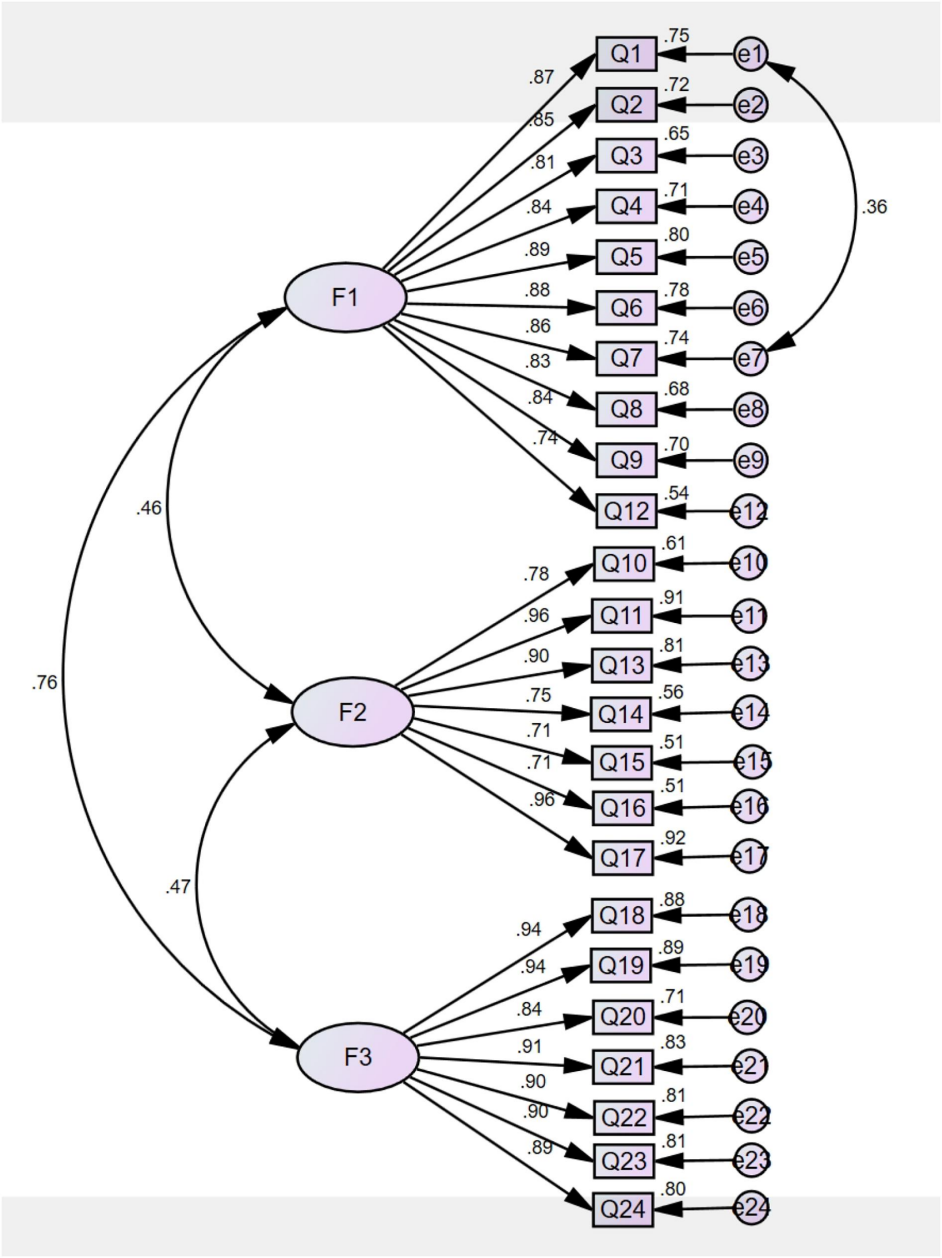
Standardized three-factor structural model of the EBC-ES (*n*_2_ = 337). F1(Treatment-related behaviors, ten items), F2(Impact-related behaviors, seven items), F3(Symptom-related behaviors, seven items).

Additionally, standardized factor loadings (Figure 2) for the EBC-ES ranged from 0.71–0.96 (all *p* < 0.01), surpassing the recommended cutoff of 0.70. This confirms strong item-construct relationships, demonstrating robust construct validity.

#### Convergent validity and discriminant validity

Table 6 further validates the robust psychometric properties of the Chinese EBC-ES. The validity of the model was comprehensively evaluated using multiple psychometric indices, including CR, AVE, MSV, Max R(H), and the HTMT. The results demonstrated strong internal consistency, excellent convergent validity, discriminant validity, and good overall model fit. Specifically, the CR values for all dimensions exceeded the 0.7 threshold, with values of 0.960 (treatment-related behaviors dimension), 0.939 (impact-related behaviors dimension), and 0.970 (symptoms-related behaviors dimension), confirming robust scale reliability.

**Table 6:** Construct validity analysis of the Chinese revision of EBC-ES.

Factor	CR	AVE	MSV	Max R(H)	HTMT analysis		
					F1	F2	F3
F1	0.960	0.708	0.570	0.960	-	0.465	0.763
F2	0.939	0.689	0.221	0.939	0.465	-	0.476
F3	0.970	0.820	0.570	0.969	0.763	0.476	-

CR, composite reliability; AVE, average variance extracted; MSV, maximum shared variance; Max R (H), maximum reliability; HTMT, heterotrait-monotrait ratio.

Additionally, convergent validity was further supported by AVE values of 0.708, 0.689, and 0.820 for these dimensions, all surpassing the recommended threshold of 0.5. Furthermore, discriminant validity was confirmed, as all AVE values surpassed their corresponding MSV values (0.570, 0.221, and 0.570). The Max R (H) values for the respective dimensions were 0.960, 0.939, and 0.969, respectively, which indicate high accuracy and consistency within the model. The HTMT values for these dimensions were 0.465, 0.763, and 0.476, respectively, providing additional support the model's discriminant validity.

#### Content validity

The Chinese EBC-ES underwent rigorous content validation by a six-expert panel (four medical professionals, one psychologist, and one scale development specialist). Excellent content validity was demonstrated, with I-CVI of 0.83–1.00 and S-CVI/Ave of 0.96.

#### Criterion validity

Criterion validity of the Chinese EBC-ES was assessed against the ECBI-IS scale. Both scales showed normally distributed total/item scores (all *p* > 0.05), supporting the use of Pearson correlation analysis. Significant positive convergence was found for total scores (*r* = 0.409, *p* < 0.001), confirming criterion validity in assessing child AD- specific behavioral problems. Detailed results are presented in Table 7.

**Table 7:** Pearson correlations between the Chinese version of EBC-ES and ECBI-IS

Classify	EBC-ES	Factor 1	Factor 2	Factor 3
Factor 1	0.968**	-	-	-
Factor 2	0.951**	0.861**	-	-
Factor 3	0.945**	0.866**	0.852**	-
ECBI Intensity	0.409**	0.422**	0.397**	0.344**

- Not available.

** Signifcant correlation at the 0.001 level (two-sided).

### Reliability analysis

#### Internal consistency reliability

The Chinese EBC—ES Scale exhibited excellent internal consistency, with a Cronbach's α coefficient of 0.968 for the total scale. Specifically, the three latent dimensions demonstrated excellent internal consistency, with Cronbach's α coefficients of 0.982, 0.837, and 0.992, respectively. Additionally, the overall McDonald's Omega coefficient was 0.987, with factor-specific coefficients of 0.982, 0.835, and 0.992. All these coefficients exceeded the commonly accepted minimum threshold of 0.7, indicating robust internal consistency. Furthermore, the split-half reliability coefficient was 0.895, further indicating the satisfactory reliability of the scale. These results are summarized in Table 8.

**Table 8:** Coefficient of Chinese version of EBC-ES.

Classify	Cronbach alpha coefficient	McDonald's Omega coefficient	Split-half reliability
The total sample	0.968	0.987	0.895
F1	0.982	0.982	0.977
F2	0.837	0.835	0.824
F3	0.992	0.992	0.974

#### Test-retest reliability

A randomly selected subsample of participants (*n* = 50) completed the retest of the Chinese version of the EBC-ES following a 2-week interval. Both baseline and follow-up scores followed normal distribution (all *p* > 0.05), thereby justifying the application of Pearson's correlation coefficient analysis. The scale demonstrated excellent test-retest reliability, with a correlation coefficient of *r* = 0.969 (*p* < 0.001). This value significantly exceeded the commonly accepted clinical threshold of 0.7 for temporal stability, indicating robust consistency of the scale over time.

## Discussion

Childhood behavioral challenges specific to AD significantly impact treatment outcomes. However, existing research has been limited by generic behavioral assessments that fail to capture AD-specific challenges, such as sleep disturbances, treatment adherence resistance, and scratching compulsions. These behaviors substantially impair parental management efficacy. The EBC is the first validated instrument designed to assess psychological characteristics associated with AD-specific behavioral problems in children. Therefore, cross-cultural validation is required to establish its generalizability and clinical applicability. The primary objective of this research was to cross-culturally adapt the EBC-ES for Chinese populations and validate its psychometric properties through comprehensive reliability and validity testing. Through rigorous cultural adaptation and psychometric validation, a 24-item Chinese EBC-ES was established. The Chinese version retained the original scale's three-factor structure, confirming cross-cultural consistency. Critically, it demonstrated robust psychometric properties, including test-retest reliability, construct validity, content validity, and discriminant validity. These results support its applicability for evaluating AD-specific behavioral problems in pediatric clinical and research settings.

The Chinese EBC-ES demonstrated excellent internal consistency and strong test-retest reliability, with all measured metrics surpassing established thresholds for clinical and research application. Notably, the scale's overall reliability was comparable to, and in certain aspects numerically exceeded, that of the original instrument. This high degree of reliability was consistently observed across all three subscales (factors), confirming the temporal stability and robust psychometric properties of the scale for measuring AD-specific behaviors over time.

The validity of the Chinese EBC-ES was rigorously established across multiple psychometric domains. The scale demonstrated excellent content validity, confirming its relevance and accuracy for measuring AD-specific behaviors in the target cultural context. Furthermore, it exhibited strong discriminant and construct validity, with all statistical indices meeting or exceeding recommended psychometric standards. The factor structure was robust, with all items loading significantly onto their hypothesized factors, thereby confirming the theoretical underpinnings of the instrument.

Both exploratory and confirmatory factor analyses provided strong support for the three-factor structure of the Chinese EBC-ES. The factor solution demonstrated excellent explanatory power and all items loaded significantly onto their intended factors, confirming the structural robustness of the adapted scale. While the original three-factor model was preserved, cross-cultural psychometric variations were identified, including the exclusion of item 15 from factor 2 due to its inadequate loading, as well as observed differences in factor loading patterns. These observed discrepancies may be attributed to a combination of factors, with the case of item 15 offering a clear example of cross-cultural contextual influences. The removal of this item (“Frequently visits the nurse at school”) was necessitated by profound cross-cultural disparities in school healthcare systems and parental health-seeking behaviors between the Australian and Chinese contexts, rather than by translation inaccuracies. This conclusion is supported by two key contextual factors. First, fundamental structural differences exist between the school-based healthcare systems of the two countries. In contrast to the Australian educational framework, where dedicated school nurses are systematically integrated, the Chinese educational environment predominantly lacks specialized, permanent nursing professionals. Routine health concerns are typically managed by classroom teachers or temporary medical personnel. Consequently, the concept of “visiting the school nurse” is culturally unfamiliar to most Chinese children, rendering this measurement item methodologically inapplicable for the target population. Second, parental health-seeking behaviors in China demonstrate a strong preference for formal medical institutions over school-based resources, which are often perceived as having limited clinical authority. This perspective significantly reduces the likelihood of parents relying on school services for managing chronic conditions such as atopic dermatitis. Instead, management typically occurs through home-based care or visits to specialized hospitals. Beyond this specific instance, broader reasons for the psychometric variations likely include: First, cross-cultural disparities in perceptions of childhood AD-specific behavioral problems, attributable to distinct cultural backgrounds, may constitute the primary cause of variations between the adapted and original scale versions. Second, imperfect conceptual equivalence during translation—including semantic, idiomatic, and experiential discrepancies—may have compromised the scale's structural validity. Third, variations in sample characteristics may also lead to divergent psychometric properties. In this study, we enrolled a large clinical sample from a premier tertiary care center, with a sample size significantly exceeding that of the original validation study. This robust sampling strategy not only enhances statistical power but also enables a more rigorous psychometric evaluation.

To our knowledge, this is the first study in China and second globally to assess AD-specific behaviors problems in pediatric populations using the EBC-ES. The Chinese adaptation yielded a total score that was marginally lower than, but not statistically different from, the original Australian version's score. This observed discrepancy may reflect culturally mediated reporting biases, wherein Chinese parents—influenced by collectivist values, Confucian emphasis on familial harmony, and traditional child-rearing philosophies—may exhibit systematically lower reporting frequencies of child behavioral problems compared to Western counterparts. The comparative prevalence of AD-specific behavioral problems between Chinese and Australian pediatric populations remains inconclusive, as current evidence lacks directly comparable multicenter studies with standardized diagnostic instruments and matched demographic characteristics. Most notably, our study confirmed that symptom-related behaviors constituted the most impactful domain of behavioral manifestations, with scores significantly surpassing other clinical areas.

Our results corroborate the existing literature, confirming that skin barrier dysfunction and itch-induced sleep disturbances represent the most prevalent behavioral manifestations in pediatric AD (15). This finding is further strengthened by the work of Moraes MM et al. (56) and is supported by previous clinical evidence (56–58). Our study identifies significant clinical and sociodemographic determinants of AD-specific behavioral symptoms within a Chinese pediatric population.

Our findings regarding criterion validity revealed a moderate correlation between the EBC-ES and the ECBI-IS. Crucially, this magnitude of correlation was expected and strengthens the validity argument for the EBC-ES. Rather than being a limitation, this finding provides strong evidence of discriminant validity, by demonstrating that the EBC-ES measures a construct that is related to, yet distinct from, general behavioral problems. While generic behavioral scales like the ECBI-IS fail to fully capture core AD-specific manifestations (e.g., scratching, sleep disruption, and sensory avoidanc), the EBC-ES is specifically designed to assess these unique behavioral challenges. This moderate correlation, therefore, underscores the unique value of our AD-specific tool in capturing a domain that was previously unmeasured. The stronger correlations observed between the factors within the EBC-ES itself further support its internal cohesion as a unified measure of AD-specific behaviors. The development of the EBC-ES was motivated by the critical gap in available tools for assessing AD-specific behaviors in children. Future studies would benefit from comparing the EBC-ES with other condition-specific measures, should they become available, to further triangulate its validity.

Our analysis identified several key sociodemographic risk factors for AD-specific behavioral problems in Chinese children, including male sex, maternal-reported parenting roles, and lower household income. Notably, these determinants extend beyond the domains captured by the original scale, highlighting a culturally-specific context that necessitates future validation. Consistent with Kisieliene et al.'s report on gender-specific AD patterns (59), we observed notably higher behavioral problem scores among male patients compared to females. Specifically, the observed gender disparity in clinical outcomes may be mediated by differences in treatment adherence, as female patients demonstrated better compliance with dermatologic regimens. This finding aligns with the report by Haruka et al., who reported superior treatment adherence among women in chronic dermatological conditions and highlighted “appearance-related concerns” and “societal role expectations” as potential mediating factors (60). Such improved adherence—including regular moisturizing and avoidance of irritants—is known to alleviate AD-related symptoms (e.g., pruritus and xerosis), thereby reducing subsequent behavioral sequelae (61). Conversely, male patients demonstrated lower adherence, potentially influenced by factors such as limited AD-specific health literacy and gender-differentiated socialization. Thus, these psychosocial factors may interact with biological sex differences, possibly exacerbating AD severity in males (62). Importantly, while these cultural and psychosocial pathways are theoretically plausible, they remain speculative and require empirical validation through future research specifically designed to test these mechanisms. Moreover, maternal-reported scores on the EBC-ES were consistently higher than paternal reports. This discrepancy may be explained by mothers' primary caregiver role, which affords greater exposure and potentially enhances symptom recognition (63, 64). Alternatively, context-dependent behavioral variations may be operative, as children often exhibit increased compliance during father-child interactions, thereby reducing paternal reports of disruptive behaviors (65–67). Furthermore, our analysis revealed a clear inverse relationship between socioeconomic status (SES) and AD-specific behavioral problem scores. Children from lower-income households exhibited markedly higher scores, empirically confirming the SES-behavioral morbidity link previously established in the literature (68). This association is likely mediated by a bidirectional dynamic: poverty-related adversities (e.g., financial strain, psychosocial stress) can directly impair disease management efficacy (69) while concurrently undermining parental coping capacity and a child's neurodevelopmental resilience (70). These interconnected pathways collectively sustain a self-perpetuating cycle that exacerbates behavioral morbidity (71).

Finally, accumulating evidence underscores the pivotal role of integrating behavioral and psychological interventions into standard AD care. Structured therapies, such as habit-reversal therapy (HRT) and cognitive-behavioral therapy (CBT), represent a promising approach by simultaneously targeting core pathophysiological mechanisms like the itch-scratch cycle and mitigating associated psychosocial factors (72, 73). To objectively evaluate the benefits of such interventions, future longitudinal studies building on our institution's validated methodologies for patient-reported outcomes (74) will be crucial. These studies should track dynamic changes in EBC-ES scores throughout the disease trajectory and in response to treatment.

## Limitations

To our knowledge, this represents the first translation and validation of the EBC-ES in China, though several limitations should be acknowledged. First, the single-center, tertiary hospital setting in Northeast China represents a key study limitation, likely resulting in an overrepresentation of moderate-to-severe AD cases and limiting the generalizability of the findings to community-based populations and individuals with milder disease. This selection bias is inherent to the recruitment strategy. Future multi-center studies spanning diverse healthcare levels (e.g., primary care clinics, community health centers) are essential to enhance external validity and better reflect the broader AD population spectrum. Second, the exclusive reliance on guardian-reported outcomes, without corroboration through clinician assessment, risks introducing reporting bias due to potential discrepancies between caregiver perceptions and objective clinical evaluations. Incorporating standardized dermatologist-rated severity assessments (e.g., EASI, SCORAD) in future validation work is crucial to improve measurement accuracy and strengthen the scale's psychometric robustness. Third, as a cross-sectional validation study, our analysis did not assess the responsiveness of the EBC-ES to clinical change over time. This limits its potential application in intervention studies. Future longitudinal studies in interventional settings are now needed to evaluate the sensitivity of the EBC-ES scores to change over time, for instance, following standard-of-care treatment or novel therapies. This will be crucial for establishing its role as a primary outcome measure in clinical trials. Furthermore, our analysis did not account for potentially important confounding factors, such as family psychosocial support resources or treatment adherence levels. These factors may significantly influence both AD severity trajectories and caregiver-reported behavioral manifestations.

## Conclusions

This study establishes the Chinese Eczema Behavior Checklist Extent Scale (EBC-ES) as a psychometrically robust tool for identifying AD-specific behavioral problems in pediatric populations. To disrupt the cycle of disease exacerbation linked to socioeconomic stressors, we advocate for integrated public health strategies that: (1) implement community-based psychosocial support programs targeting high-risk families, and (2) scale structured educational initiatives teaching adaptive coping mechanisms across healthcare tiers. The validated scale serv es as a precision public health screening instrument, enabling efficient targeting of limited resources to children with greatest need—ultimately reducing long-term psychosocial burden and advancing health equity in AD management.

## Data Availability

The original contributions presented in the study are included in the article/Supplementary Material, further inquiries can be directed to the corresponding author.
